# Plant Aspartic Proteases for Industrial Applications: Thistle Get Better

**DOI:** 10.3390/plants9020147

**Published:** 2020-01-23

**Authors:** André Folgado, Rita Abranches

**Affiliations:** Plant Cell Biology Laboratory, Instituto de Tecnologia Química e Biológica António Xavier (ITQB NOVA), Universidade Nova de Lisboa, 2780-157 Oeiras, Portugal; afolgado@itqb.unl.pt

**Keywords:** cardoon, cardosin, recombinant protein, molecular farming, milk clotting, cheese

## Abstract

Plant proteases have a number of applications in industrial processes including cheese manufacturing. The flower of the cardoon plant (*Cynara cardunculus* L.) is traditionally used as a milk-clotting agent in protected designation of origin cheeses made from goat and sheep milk. Plant-derived rennets are of particular importance to consumers who wish to eat cheeses that are produced without harming any animals. In this review, we have highlighted the importance of plant proteases, particularly aspartic proteases, in industrial processes, as well as exploring more fundamental aspects of their synthesis. We have also reviewed and discussed the production of these enzymes using sustainable and cost-effective alternative platforms.

## 1. Introduction

Plant proteases constitute one of the main groups of plant proteins with important industrial applications, including in fields such as detergents, the food industry, clothing, and pharmaceuticals [1]. The main application of plant aspartic proteases (APs) is in the dairy industry as milk-clotting agents during cheese manufacturing. These cheeses usually have a regional character and are representative of small-scale production in rural areas. Most of these cheeses have a protected designation of origin that contributes to their valorization and adds market value. Although these cheeses still represent market niches, they are an important source of income for local communities and have a high impact on the preservation of specific regional breeds of sheep and goat [2]. The use of plant APs as rennet in cheese manufacturing is mainly driven by the shortage in animal rennet, which is extracted from the stomach of young calves. The main enzymes present in the bovine stomach are chymosin and pepsin and their proportion is age-dependent, normally 88–94% chymosin and 6–12% pepsin at a young age [3]. In adult animals, these proportions are reversed to reflect the different diet in adulthood [4]. Rennet is extracted from the stomachs of young calves as the ratio of chymosin to pepsin favors effective milk-clotting activity over excessive proteolytic activity. The high demand for clotting enzymes and a shortage of supply has resulted in a search for alternative sources of milk-clotting enzymes. Technically, any protease that displays proteolytic activity towards the caseins that can structurally destabilize the casein micelle has the potential to be used as a milk-clotting agent. However, the ability of a protease to be used for milk-clotting also depends on other factors that will influence both curd formation and yield. The suitability of an enzyme for milk-clotting is determined by the ratio between its milk-clotting activity (MCA) and nonspecific proteolytic activity (PA). A suitable enzyme therefore has a high MCA/PA ratio, which means high specificity for κ-casein and low non-specific proteolytic activity [5]. The low MCA/PA ratio characteristic of many different plant proteases has been highlighted as one of the main factors preventing their application as milk-clotting agents on an industrial scale [6].

Although many plant proteases demonstrate an ability for milk-clotting, the actual application of the majority of these proteases is still experimental or on an artisanal scale [5]. The APs from the flower of *Cynara cardunculus* L., also known as cardoon or thistle flower, are currently the only suitable vegetarian rennet for cheese manufacturing and the only one with applicability for cheese production [7]. However, these APs can only be used on a semi-industrial scale, which is mainly due to their higher proteolytic activity when compared with enzymes from animal sources. This has a direct impact on the final product yield, hampering application at industrial scales. Despite the fact that thistle APs display higher proteolytic activity than their animal counterparts, a market niche has been found for cheeses produced with raw milk from sheep and goats, which has led to the production of distinct and highly valued products.

**Figure 1:**
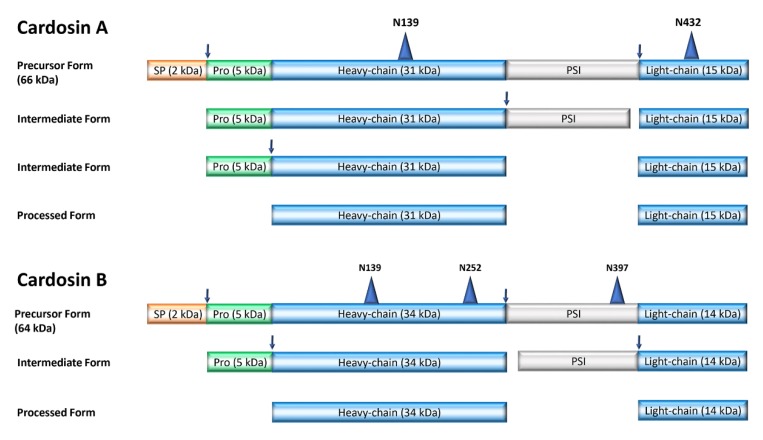
Schematic representation of the processing steps of cardosin A and cardosin B. SP: signal peptide; Pro: prosegment; PSI: plant-specific insert. Arrows mark the cleavage sites during processing. Triangles indicate glycosylation sites. Based on References [14,15,16] and UniProt KB accession Q9XFX3 and Q9XFX4.

Most plant aspartic proteases belong to the A1 family and can be divided into typical, nucellin-like, and atypical APs (for a recent review see Reference [8]). The APs found in thistle flowers are typical APs and are, as most plant APs, characterized by the presence of two aspartic acid residues in the two conserved catalytic motifs formed by Asp–Thr/Ser–Gly (DT/SG). They have optimal activity under acidic conditions and are sensitive to pepstatin A, a hexapeptide from *Streptomyces* that inhibits AP activity [9]. They are synthesized as zymogens, a single-chain and inactive form of the protein that contains several elements: a signal peptide sequence that directs the protein to the secretory pathway, a prosegment that is involved in both activation and protein folding, and a 100 amino acid segment that links the N- and C-termini of the APs. This 100 amino acid sequence is specific to plant APs and thus denominated plant specific insert (PSI) [10] (see Figure 1). Plant APs undergo successive activation processes in which segments of the sequence are removed in order to make the APs active. Among these removed parts are the prosegment and the PSI [11]. The role of the PSI is not yet clear, but some reports suggest a putative role in protein trafficking and also in plant defense mechanisms [12,13]. 

In this review, we focused on the general use of thistle APs as clotting agents for the dairy industry, the genetic variability among thistle populations, its effect on flower AP content, and the impact on cheese manufacturing. We have also discussed the possible trafficking routes of cardosins within plant cells, the different aspects involved in expression and activation of plant Aps, and their implications for the expression and production of thistle APs in alternative production systems, such as bacteria, yeast, and plant cells.

## 2. The Thistle Flower and the Impact of Its Variability on Milk-Clotting Activity

The use of the cardoon flower as a milk-clotting agent in traditional cheese manufacturing is ancient, and dates back as far as Roman times [14]. In contrast to artichokes, thistle cultures were never subjected to a breeding program. Thus, the commercial exploitation of the flowers is based on the harvesting of flowers from wild cardoon populations (i.e., not grown under controlled conditions). This has been the subject in recent years of several research projects aimed at understanding the level of variability among cardoon populations and its impact on enzymatic stability and cheese manufacturing.

The study of morphological characteristics has shown a great biodiversity among different cardoon populations and confirmed the wide genetic variability that is a characteristic of this species [15]. The variability of thistle populations is not limited to morphological characteristics; it has also been observed in the AP content of the flowers. The evaluation of individual genotypes showed that the observed biodiversity is also reflected in the amount and type of APs present in the flowers of each genotype, with enzymes present in some genotypes and absent in others [16]. This variability was also found to affect the milk-clotting process, as the use of flower extracts from different thistle populations displayed different milk-clotting times, which ultimately affected cheese manufacturing and the rheological and sensorial characteristics of the final product [17,18]. Thus, the use of thistle flower extracts as milk-clotting agents is dependent upon the presence and specific activities of different APs. Since the growth and harvest of the flowers are not controlled processes, factors such as the specific origin of the flowers, the tissues that are harvested, and how they are dried contribute to a greater variability in AP content of thistle flower (prior to extraction). This variability can result in the preparation of enzymatic extracts with different clotting strengths and, as a result, affects the predictability and stability of the cheesemaking process [19]. These studies have demonstrated the great genetic variability that characterizes thistle populations and the way in which it influences the cheesemaking process and the quality of the final product.

A multifamily of APs can be found in the pistils of the cardoon flower and are called cyprosins and cardosins [20,21]. These enzymes are responsible for the breakdown of casein, the major milk protein. The characterization of these proteases is of great importance since they influence the specific characteristics of the cheese produced, mostly in terms of texture and flavor. A number of different APs have been identified in the cardoon flower [22,23,24], and have been found to constitute a multi-gene family [25]. To date, nine different APs have been found at the protein level, six cardosins and three cyprosins. The most studied and well characterized are cardosins A and B. Cardosins C and D were identified only at the mRNA level [25]. Most of the cardosins identified, with the exception of cardosin B, share homology with cardosin A, which hampers its isolation from the flower. Despite the large number of APs identified in the flowers of cardoon, little is known about their biological functions. The increasing expression of cyprosins during development of the flower has been associated with putative roles in reproduction or senescence [20]. Our preliminary analysis on the expression of cardosins and cyprosins genes in flowers at different developmental stages (closed, partially open, and open), confirmed the high variability of gene expression within and between genotypes (unpublished results). The expression of APs detected in other tissues led to the hypothesis that their expression, as well as protein function, may be tissue-dependent. Nonetheless, the biological role of cardosins and cyprosins remain unknown.

Within the various APs found in the thistle flower, cardosins A and B are the most extensively studied regarding proteolytic activity. The different activities of these two proteases on milk caseins are clear: cardosin A is more specific and less proteolytic than cardosin B and its activity is more similar to that of animal chymosin; conversely, cardosin B is more proteolytic and less specific than cardosin A, and its activity is more similar to that of animal pepsin [21]. The difference in enzyme activity is related to the action that these enzymes have on caseins. Milk caseins form a micellar structure consisting of α, β, and κ-caseins. α and β-caseins are composed of hydrophobic structures whereas κ-casein is composed of hydrophobic and hydrophilic structures. Thus, κ-casein is responsible for the stabilization of the micelle and causes its solubilization [26]. Milk coagulation occurs due to the destabilization of this complex through proteolytic action on κ-casein. The hydrolysis of κ-casein exposes the hydrophobic structures and promotes the binding of casein micelles to each other, forming a network that leads to the formation of the milk curd. During syneresis, which consists of the release of water from the curd, the hydrophilic structures remain in the whey, along with a large fraction of the enzymes used in this process [27]. Chymosin has a very specific activity on κ-casein, cutting only between amino acids Phe105 and Met106 of the protein. This action leads to the production of two peptides, para-κ-casein and macropeptide [28]. APs from the thistle flower also cleave at the same site [29]; however, the proteolytic activity is more extensive than chymosin [30,31,32]. This broader activity towards caseins has been suggested as a potential cause for the bitter and spicy flavors of cheeses produced with these enzymes [33]. In addition to the genetic variability already described for thistle populations, the different AP profiles found among flowers of different genotypes further increase the variability of the flower extracts used for milk-clotting. This variability influences the clotting time of the extract and contributes to batch-to-batch unpredictability.

## 3. Cellular and Intracellular Localization of Cardosins

Cardosins A and B not only have different properties, but are also found in different locations in the cardoon flower. Cardosin A was found in the vacuoles of the stigmatic papillae, while cardosin B was found in the extracellular matrix of the transmitting tissue of the flower [34,35]. This different localization contributed to the notion that cardosin B was secreted into the extracellular space, although the authors highlighted that this fact may simply be because the transmitting tissue cells do not express the specific vacuolar sorting receptors [34]. 

The study of cardosins in other tissues has shown that their expression is not limited to the flowers and that the protein processing is tissue-dependent. Both cardosins A and B were found during postembryonic development of thistle [36], in the callus of thistle tissues [37], and also accumulated in the vacuoles when expressed in heterologous systems such as *Arabidopsis thaliana* or tobacco leaves [38,39]. This different behavior led to trafficking studies to further understand the movement of cardosins within cells. 

The movement of cargo between organelles is performed by a trafficking system composed of vesicular and tubular membranous carriers. The transport is sequential and consists of cargo selection followed by formation of vesicles from the donor membrane, transport of vesicles, the tethering of vesicles to the target membrane, and finally fusion of the vesicle with the target membrane [40]. All of these steps are regulated by specific machinery that involves coat protein complexes and Rab GTPases. Studies on the cardosin-trafficking pathway have revealed that both cardosins A and B leave the endoplasmic reticulum (ER) and travel to the Golgi in a RAB-D2a (RABD2=RAB1)-dependent manner. From the Golgi, both proteins are transported to the vacuole via a RAB-F2b (RABF2=RAB5)-dependent pathway [12,39]. Although these proteins move through the Golgi during their processing, they showed differences in glycosylation patterns. Cardosin A was shown to be endo-H-resistant while cardosin B was sensitive, indicating that cardosin A possesses complex glycans in its glycosylation profile. Crystallography studies on cardosin A confirmed the complex glycosylation pattern [38,41], while cardosin B glycosylation was found to consist of mainly high-mannose type glycans [39]. The different glycosylation pattern for cardosin B was justified by either a shorter passage through the Golgi or inaccessibility of the glycosylation sites in the folded protein to glycan-processing enzymes [39]. Furthermore, these proteins showed differences in their activation processes. Cardosin A trafficking studies have revealed that the protein is only activated after reaching the vacuole. Transient expression studies of cardosin A in tobacco leaves detected intermediary forms of cardosin A, corresponding to the activation steps that the enzyme passes through; however, its active form was only found in the vacuole [38]. In the case of cardosin B, activation seems to occur much earlier, since the protein was mostly found in its active form, with no intermediary stages detected. This suggests an early and very fast activation process, possibly occurring as early as the ER, although the precise location and activation process are still unknown [39]. More specific studies using inhibition of transit from the ER to the Golgi showed that the trafficking regulatory domains of these proteins are highly complex [12]. Differences in PSI glycosylation affect vacuolar trafficking; while the PSI from cardosin A enables trafficking to the vacuole in a COPII-independent manner, the PSI from cardosin B coordinates vacuolar sorting in a COPII-dependent manner. Moreover, these studies also showed that there is a hierarchy at the vacuolar sorting signal (VSS) level and, in the case of cardosin A, the C-terminal peptide was shown to be the dominant VSS over the PSI segment, controlling the trafficking pathway when both signals are present and the transit from the ER to the Golgi is blocked. Similar results were obtained for phytepsin, an aspartic protease from barley, in which the PSI was also found to be responsible for COPII-dependent sorting. As for cardosin B, the PSI from phytepsin is also glycosylated, which supports the role of glycosylation as a regulatory mechanism in AP-trafficking pathways. However, in contrast to cardosin B, removal of the PSI segment from phytepsin led to its secretion from the cell, even when the COPII pathway was blocked, suggesting a different way for this AP to leave the ER [42]. Different behaviors were also found for APs in soy, with different APs showing different responses to PSI truncation. SoyAP1 vacuolar sorting was unaffected by the removal of the PSI sequence as it was still able to reach the vacuole, suggesting that another VSS is present in the sequence. Meanwhile, soyAP2 remained in the ER following PSI truncation [43]. Although these studies were not as exhaustive as those performed for cardosins, they showed that plant APs differ in their sorting strategies. These differences may be associated with the tissue-dependent expression of each AP as well as the cell’s need for regulatory plasticity when facing environmental stresses or developmental processes. This knowledge of trafficking pathways and their sorting determinants is crucial for the development of alternative production platforms for these APs, since they will greatly influence the ability of the cells to secrete the enzymes to the culture medium.

## 4. Production of Thistle APs Using Alternative Platforms

Due to their importance to the cheese sector, the industrial production of APs from thistle has been a matter of great interest over the years. This has led to several studies on the expression of these APs in heterologous systems such as yeast, bacteria, and plant cell cultures (Table 1).

In this vein, the expression of cyprosin B was studied in *Pichia pastoris* and *Saccharomyces cerevisiae*. Expression in *P. pastoris* resulted in accumulation of the processed form in the culture medium; however, the enzyme processing led to an incomplete removal of the PSI, resulting in a form of the protein with both heavy and light chains held together by disulfide bonds, which differs from the cyprosin B found in the thistle flower. This led to differences in structure and catalytic efficiency [46]. More recently, an AP from grasshopper (*Galium verum* L.) was expressed and produced in *P. pastoris* [49]. In this work, the authors found similar results as for cyprosin B: the protein was found in the culture medium in its processed form with both chains held together by disulfide bonds. However, a deeper analysis throughout the growth curve revealed that the protein was secreted into the culture medium in its unprocessed form, and further processing occurred due to changes in the pH of the culture medium. At some point the medium became acidic, leading to processing of the unprocessed secreted form [49]. Expression in *Saccharomyces cerevisiae* showed different results: in this system, recombinant cyprosin B was in part secreted into the culture medium as a mixture of active and inactive forms; however, the secretion process was limited, and a significant part was retained within the cell. This makes the fermentation process dependent on biomass production and limits its industrial use [47]. These studies emphasized how the forms in which these enzymes are expressed are highly dependent on the expression system used. In fact, one of the problems associated with the production of thistle APs in heterologous systems is their different behaviors in these systems. A system that works for one enzyme may not work for another. 

Almeida and coworkers [44] developed a strategy that allowed for the production and secretion of synthetic cardosin B (VRen) in *Kluyveromyces lactis* using protein engineering. In this work, the alterations introduced into the structure of the enzyme included the removal of the PSI and the union of the two subunits through a glycine linker. This strategy allowed for the synthetic form of cardosin B to be produced and secreted into the culture medium in its inactive form, requiring a subsequent activation step by acidic pH to obtain the functional enzyme [44]. This strategy had already been used in studies involving the expression of recombinant cardosin A in *Escherichia coli*, in which the removal of PSI led to the formation of a single chain protein and the only step during activation was the removal of the prosegment. The single-chain recombinant cardosin A was active, with slight differences in its specificity for amino acids in the P1′ position when compared to normal recombinant cardosin A [11]. The same strategy was used for the production of recombinant cardosin B, which also exhibited altered specificity [45]. The single-chain form showed a more stringent specificity and lower catalytic efficiency in comparison with the native cardosin B. The authors explained this difference as being due to possible diminished flexibility of the enzyme in the C-terminal region due to the direct fusion of the heavy and light chains [45]. This fact is very important and could have a great impact in the successful application of synthetic cardosin B in cheese manufacturing at industrial scale. Cardosin A is often highlighted as being more suitable for promoting milk-clotting due to its higher specificity and lower proteolytic activity when compared to cardosin B. However, the amount of cardosin A needed for milk-clotting has been found to be 10-fold higher than that of cardosin B, i.e., the specific activity of cardosin A is lower than cardosin B [32]. The practical application of the extract enriched with the alternative form of cardosin B, VRen, has also been demonstrated in cheese production [44]. These reports demonstrate the possibility of large-scale production of synthetic cardosin B and the enormous potential for innovation that exists with the application of protein engineering in this area. Due to its more specific proteolytic activity, synthetic cardosin B has the potential to be the first of the cardosins to become viable at an industrial scale. Nevertheless, and despite this major breakthrough, these results highlight the difficulties of developing a biotechnological solution that is suitable for the commercial exploitation of all plant APs.

## 5. Molecular Farming of Aspartic Proteases

The search for alternative sources of plant APs has not been confined to yeast or bacteria. The first system to be explored was the use of tissue culture from the thistle plant itself. Figueiredo and colleagues established cultures of suspension cells from thistle leaves with the intention of using these undifferentiated cells as an alternative source of thistle PAs in the future [50]. In this work, undifferentiated cell cultures were successfully established, and the culture medium used in their induction was optimized. Later, Lima Costa and coworkers [51] studied the growth of these cultures in a continuous system in a chemostat and measured both proteolytic activity and phenol production, comparing the production of chemostat cultures to that of batch cultures. Chemostat cultures demonstrated a higher total proteolytic activity without the production of phenol compared to batch cultures. However, the protease class was not determined and, as such, it was not possible to establish whether the measured proteolytic activity was due to AP activity [51]. These cultures were further characterized in terms of AP expression. Oliveira and coworkers studied the expression of cardosins A and B in thistle callus and demonstrated the *de novo* production of these cardosins, which were shown to accumulate in the ER in an unprocessed state. This work demonstrated that these undifferentiated cells were able to produce cardosins, but their processing was different from that found in the flowers [37]. Although the results showed that the use of thistle callus cultures as an alternative source of cardosins might not be viable, it provided important fundamental data relating to AP processing in the system. These data support the use of genetic engineering to improve the system to become a production platform for thistle APs.

In another study, Sampaio and coworkers [48] produced recombinant cyprosin B by genetic transformation of thistle callus in order to overexpress the protein, and subsequently characterized the production in a bioreactor. The authors demonstrated the ability of this system to produce cyprosin B in its active form, although it was accumulated inside the cells and not secreted into the culture medium. Although it was possible to purify the protein, the yield was still a limiting factor for the adoption of this methodology on an industrial scale [48]. More recently, Wei and coworkers produced an active chymosin in tobacco plants, reporting a production of 83.5 ng/g of fresh weight tobacco leaves [52]. Recently, our group established hairy root cultures from thistle explants and characterized these cultures in terms of their protease content. Proteases from the serine, cysteine, and aspartic classes were identified. Among the APs, expression of cardosin A and D genes was detected, and cardosin A protein was found in its active form in hairy root protein extracts [53]. These examples represent a promising start for the field of molecular farming as applied to the production of APs. Although these platforms are still far from being established as cost-effective alternative sources of APs for milk-clotting, they show the potential for plant systems in the production of active APs. 

## 6. Challenges and Opportunities

Despite their abundance in nature and their economic importance, plant APs are still poorly studied with regard to their sorting mechanisms, functions, and structures. Of the plant APs that have been investigated, cardosins are among the best-studied for biotechnological applications. As discussed, the production of plant APs using alternative platforms is highly influenced by the expression platform used and the form of the protein produced. Production of plant APs in plant-based platforms may become the solution that allows for the industrial exploitation of APs. Plant-based systems offer the same structural and basal mechanisms that are present in nature for the expression and processing of APs, thus making AP production similar to that occurring in nature. This fact probably explains why cyprosin B was purified in its active form when expressed in thistle cell suspension cultures. Our own research has already given the same results for the expression of cardosin B in BY2 cell suspension cultures (Folgado et al., in preparation). This is an advantage with regard to other expression platforms and prevents an activation step during downstream processing. However, the similarity of plant-based production platforms also leads to accumulation of APs in the vacuoles of cells, impairing secretion of the protein to the culture medium and hampering scalability. The protein engineering strategies applied to cardosin B, namely removal of the PSI segment, enabled the secretion of the protein in yeast. However, the same strategy applied to cardosin B expressed in BY2 was not as successful (Folgado et al., in preparation).

Collectively, the knowledge obtained to date supports the use of plant cell suspension cultures as alternative platforms for the production of thistle APs and encourages future studies to further develop these platforms. The secretion of thistle APs to the culture medium and strategies to increase production yields and also to facilitate purification processes are research fields that need to be explored in the near future in order to strengthen these platforms as sources of thistle APs. As previously stated, promotion of milk-clotting by thistle flower extracts is the result of the collective activity of all APs, which varies between different thistle populations. The development of platforms able to produce individual thistle APs would enable the use of single or combined enzymatic formulations in the cheese manufacturing process, leading to the development of new and customized products. This could also contribute to increased control of the cheese manufacturing processes using thistle APs and allow for their adoption on an industrial scale.

## Figures and Tables

**Table 1:** Thistle APs expressed in heterologous systems and their processing state in each system.

Thistle AP	Organism	Processing	Reference
Cardosin A	*Escherichia coli*	Inactive	[11]
Cardosin A	*Nicotiana tabacum* *Arabidopsis thaliana*	Active	[38]
Cardosin B	*Kluyveromyces lactis*	Inactive	[44,45]
Cardosin B	*Nicotiana tabacum* *Arabidopsis thaliana*	Active	[39]
Cardosin B	Tobacco BY2 cell suspension cultures	Active	Folgado et al. (in preparation)
Cyprosin B	*Pichia pastoris*	Inactive	[46]
Cyprosin B	*Saccharomyces cerevisiae*	Inactive/active	[47]
Cyprosin B	*Cynara cardunculus* L. cell suspension cultures	Active	[48]
